# Microglia activation in periplaque white matter in multiple sclerosis depends on age and lesion type, but does not correlate with oligodendroglial loss

**DOI:** 10.1007/s00401-023-02645-2

**Published:** 2023-10-28

**Authors:** Wiebke Kessler, Christian Thomas, Tanja Kuhlmann

**Affiliations:** Institute of Neuropathology, University Hospital Münster, Pottkamp 2, 48149 Münster, Germany

**Keywords:** Multiple sclerosis, Microglia, Periplaque white matter, Mixed active/inactive lesions, Aging

## Abstract

Multiple sclerosis (MS) is the most frequent inflammatory and demyelinating disease of the CNS. The disease course in MS is highly variable and driven by a combination of relapse-driven disease activity and relapse-independent disease progression. The formation of new focal demyelinating lesions is associated with clinical relapses; however, the pathological mechanisms driving disease progression are less well understood. Current concepts suggest that ongoing focal and diffuse inflammation within the CNS in combination with an age-associated failure of compensatory and repair mechanisms contribute to disease progression. The aim of our study was to characterize the diffuse microglia activation in periplaque white matter (PPWM) of MS patients, to identify factors modulating its extent and to determine its potential correlation with loss or preservation of oligodendrocytes. We analyzed microglial and oligodendroglial numbers in PPWM in a cohort of 96 tissue blocks from 32 MS patients containing 100 lesions as well as a control cohort (*n* = 37). Microglia activation in PPWM was dependent on patient age, proximity to lesion, lesion type, and to a lesser degree on sex. Oligodendrocyte numbers were decreased in PPWM; however, increased microglia densities did not correlate with lower oligodendroglial cell counts, indicating that diffuse microglia activation is not sufficient to drive oligodendroglial loss in PPWM. In summary, our findings support the notion of the close relationship between focal and diffuse inflammation in MS and that age is an important modulator of MS pathology.

**Supplementary Information:**

The online version contains supplementary material available at 10.1007/s00401-023-02645-2.

## Introduction

Multiple sclerosis (MS) is the most frequent demyelinating disease of the central nervous system (CNS). The histopathological hallmarks are focal inflammatory and demyelinating lesions which––depending on the lesion stage––contain variable amounts of macrophages/microglia, T cells, and B cells [25]. In the first disease phase the majority of patients are clinically characterized by a relapsing–remitting disease course. The relapses are caused by the development of new focal lesions which can occur throughout the whole CNS in gray or white matter. In the majority of patients, this relapsing–remitting disease phase progresses into a secondary progressive disease (SPMS) which is characterized by a continuous worsening of symptoms without additional relapses [28]. Interestingly, in a subset of patients with relapsing–remitting MS (RRMS) progression independent of relapse activity (PIRA), can be observed already very early during the disease course [23, 47]. However, the mechanisms driving relapse-dependent and -independent disease progression are only partly understood and may vary across patients and over time. Current concepts suggest that a combination of injury mechanisms, such as persisting focal inflammation, diffuse microglia activation in normal appearing white matter, as well as axonal and neuronal injury and (functional) loss together with the loss of compensatory mechanisms such as remyelination and brain plasticity are the drivers of disease progression in MS [26]. However, the extent of different injury and compensatory mechanisms may vary over time and between patients and it is still a challenge to dissect the relevance of the different pathologies for disease progression in the individual patient [39]. Interestingly, it seems that also aging is an important factor contributing to disease progression. In adults, older age at onset is associated with faster disability and cognitive impairment and transition from the relapsing to the progressive disease phase occurs at about the same age, regardless of the age at disease onset [7, 8]. Potential explanations for this age-associated effect on disease progression include negative effects of age on remyelination and brain plasticity, but also age-associated changes in the immune system [15, 17, 36, 38].

Whether and to which extent diffuse changes in the white matter may contribute to disease progression is still a matter of debate. Higher numbers of microglia and T cells in normal appearing white matter (NAWM) compared to control tissue samples as well as an increase in microglia numbers in NAWM of patients with SPMS compared to RRMS have been reported [20, 27, 44, 46, 52]. Interestingly, microglia activation in NAWM correlates with changes in the myelin structure. Microglia activation in non-demyelinated white matter from MS patients was associated with less compact myelin wrapping, subtle changes at the paranodes and juxtaparanodes, as well as increased intra-axonal mitochondrial densities compared to control tissue samples [13, 48]. Furthermore, microglial nodules in NAWM are already early detectable in MS and are associated with degenerating axons. However, whether the degenerating axons in NAWM are the consequence of or the cause for axonal degeneration remains unclear [43]. In recent years, bulk, single cell or single nucleus RNA sequencing contributed to the dissection of molecular signatures in the periplaque white matter (PPWM) or NAWM [1, 11, 20]. In PPWM compared to NAWM an increased infiltration of immune cells was observed. This was associated with an upregulation of genes related to the unfolded protein or stress response as well as cholesterol biosynthesis in subsets of oligodendroglial lineage cells [1]. However, nothing has been reported so far regarding the effects of lesion type, sex, or age on microglia activation in non-demyelinated white matter and whether the extent of microglia activation correlates with loss of oligodendrocytes in NAWM of MS patients.

Therefore, we performed a detailed histological study and quantified the number of microglia in 96 tissue samples from 32 patients containing 100 lesions as well as in age- and sex-matched controls (*n* = 37). Microglia activation was dependent on proximity to lesion and lesion type. Interestingly, the heterogeneity between patients was higher than the heterogeneity between lesion types, suggesting that individual factors modulate the microglia response in PPWM, and we identified age as an important potential modulator of microglial numbers. Higher microglia densities were not associated with lower numbers of oligodendrocytes in PPWM, indicating that microglia activation may not be sufficient to induce oligodendroglial loss.

## Material and methods

### Materials

This study was performed retrospectively on a collection of formalin-fixed and paraffin-embedded brain autopsy tissue specimens from 32 individuals. A total of 19 patients with 64 MS lesions from 60 tissue blocks were retrieved from the autopsy collection of the Institute of Neuropathology, University Hospital Münster, Germany. Another 13 patients with 36 lesions from 36 tissue blocks were collected from the Netherlands Brain Bank (NBB), Netherlands Institute for Neuroscience, Amsterdam (open access: https://www.brainbank.nl); all material has been collected from donors for or from whom a written informed consent for a brain autopsy and the use of the material and clinical information for research purposes had been obtained by the NBB. Additionally, 37 tissue blocks from 37 age- and sex-matched individuals from the autopsy archive of the Institute of Neuropathology in Münster were included as controls (18 males, and 21 females, average age at death 58.77 years). These control tissue samples were selected based on the absence of evidence for a neurological disease by histology and clinical history. None of the study authors was involved in decision making regarding autopsy. The Ethics Committee of the University of Münster (Ref: 2016-165-f-S, 2012-407-f-S, 2011-023-f-S) approved this study.

## Methods

### Lesion characterization

Only white matter lesions located in cerebrum (*n* = 93) or cerebellum (*n* = 7) were used for this study. Lesion types were categorized according to the classification of Kuhlmann and colleagues using immunohistochemistry (IHC) for MBP and CD68 [25]. A total of 9 active lesions, 34 mixed active/inactive lesions (subsequently named mixed lesions), and 57 inactive lesions were identified; among them 5 active, 17 mixed active/inactive, and 14 inactive lesions were from the Netherlands Brain Bank. Patient and lesion data are summarized in Table 1.

**Table 1:** Clinical details of MS patients

Total number of patients	32
Münster MS autopsies	
Total number of patients	19
Total number of lesions	64
Male:female patients	9:10
Median age ± SD	56 ± 33 years
Cause of death	
Cardiovascular failure	8
Respiratory failure	4
Sepsis	1
Euthanasia	0
Other	5
Unknown	2
Lesion type: activity	
Active	4 (6.25%)
Mixed (active/inactive)	17 (26.56%)
Inactive	43 (67.19%)
NBB MS autopsies	
Total number of patients	13
Total number of lesions	36
Male:female patients	6:7
Median age ± SD	58.62 ± 22.38 years
Median disease duration ± SD (disease duration unknown for 1 patient)	24.1 ± 16.1 years
Cause of death	
Cardiovascular failure	1
Respiratory failure	3
Sepsis	1
Euthanasia	3
Other	3
Unknown	2
Lesion type: activity	
Active	5 (13.89%)
Mixed (active/inactive)	17 (47.22%)
Inactive	14 (38.89%)

### Immunohistochemistry

For IHC tissue samples were fixed in 4% paraformaldehyde (PFA) and embedded in paraffin. For further usage tissue blocks were cut into 4-µm-thick sections and were stained for CD68, HLA-DR C3/43 (HLA-DR), Myelin binding protein (MBP), P2RY12, and TPPP/p25 (Supplementary Table S1). IHC was performed with the Dako REALTM Detection System (#K5001, Dako) and an automatic immunostainer (Autostainer Link 48, Dako) using the biotin–streptavidin method. In brief, tissue sections were deparaffinized and the intrinsic peroxidase activity was blocked by incubation with 5% H_2_O_2_ in phosphate-buffered saline (PBS). Subsequently, primary antibodies were applied as described in Supplementary Table S1. Species-specific biotinylated mouse, rat, or rabbit antibodies were applied as secondary antibodies and incubated with a streptavidin/peroxidase complex. To complete IHC, the reaction product was developed with diaminobenzidine. Tissue slides without a positive staining signal in the white matter were stained again or were excluded for further analyses. Therefore, numbers of analyzed lesions may vary between different quantifications.

### Bielschowsky’s silver staining

Deparaffinized sections were bathed in a 20% silver nitrate solution at room temperature for 20 min. 32% ammonia hydroxide was added dropwise to the silver nitrate solution. After a washing step, slides were placed in this solution in the dark for approximately 30 min till the sections turned dark brown. Afterward, slides were rinsed in a water–ammonia solution and placed in the developer working solution containing 20 ml 37% formaldehyde, 0.5 g citric acid, two drops of nitric acid, and 100 ml distilled water. Reactions were stopped by placing the slides into distilled water. In a final step, sections were placed in a 2% sodium thiosulfate solution for two minutes and washed in distilled water followed by an ascending alcohol series, xylene, and mounting.

### Quantitative analyses

**Fig.1:**
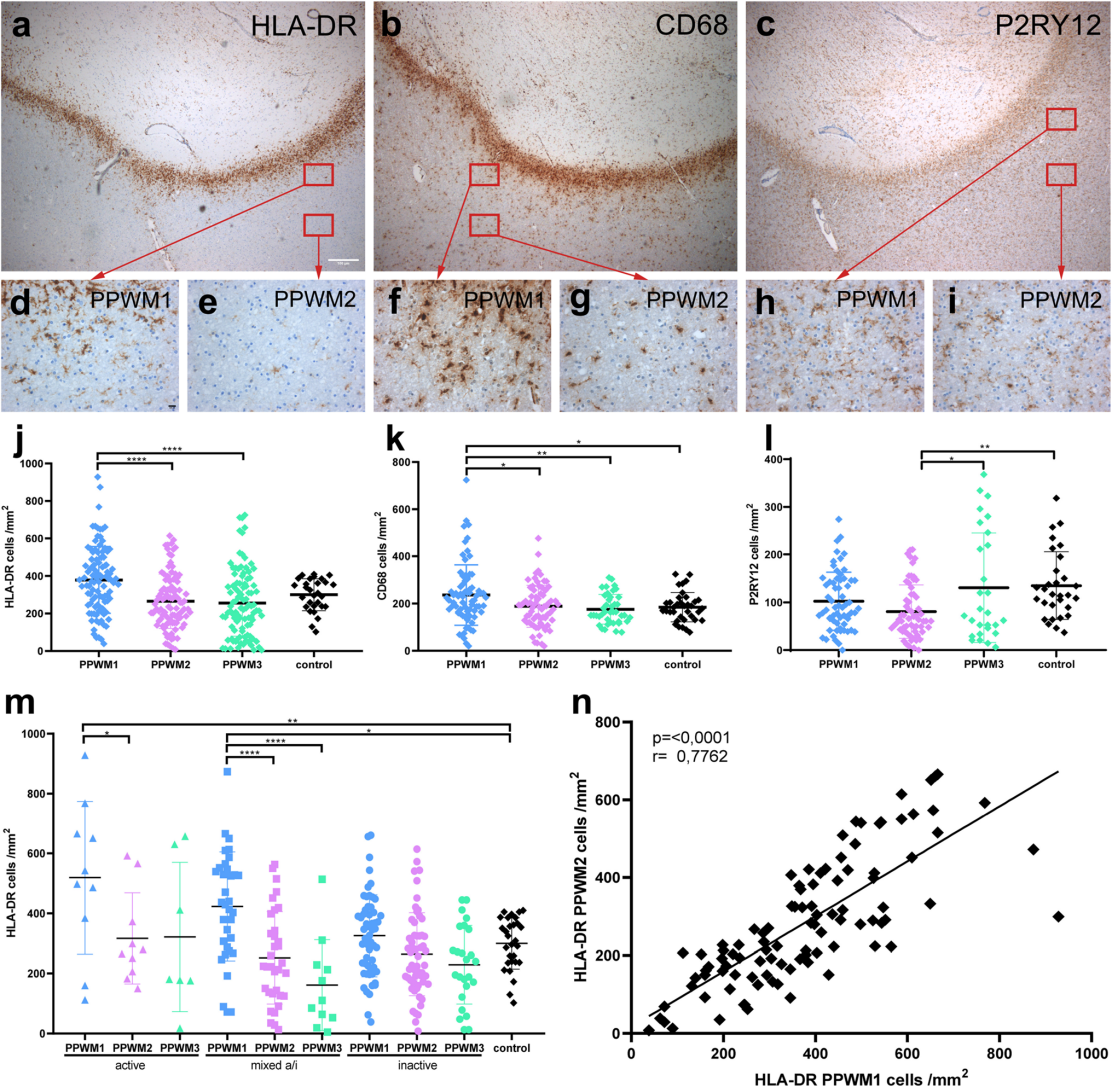
Microglia activation in periplaque white matter adjacent to different lesion types. The same lesion stained for either HLA-DR (**a**), CD68 (**b**) or P2RY12 (**c**) is depicted. Higher magnifications of periplaque white matter 1 (PPWM1) and PPWM2 stained for HLA-DR (**a**, **d**, and **e**), CD68 (**b**, **f**, and **g**), or P2RY12 (**c**, **h**, and **i**) are shown. Numbers of HLA-DR + and CD68 + microglia were increased in PPWM1 compared to PPWM2 and PPWM3 (**j** and **k**). Numbers of P2RY12 + microglia are in average lower than numbers of CD68 + and HLA-DR + microglia in PPWM1, PPWM2, and PPWM3 (**l**). Numbers of HLA-DR + microglia in PPWM1, PPWM2, and PPWM3 stratified according to lesion type. HLA-DR + microglia are significantly increased in PPWM1 around active and mixed lesions, but not inactive lesions (**m**). We observed a significant correlation in the number of HLA-DR-positive microglia in PPWM1 and PPWM2 (**n**). Graphs in j to n depict the quantifications for individual lesions. Scale bars: **a**–**c** 500 µm, **d**–**i** 50 µm, *p < 0.05, **p < 0.01, ***p < 0.001, ****p < 0.0001

In all lesion types, lesion border was defined as the border between the demyelinated or remyelinated lesion and the non-demyelinated white matter. The rims of macrophages/microglia of mixed lesions were considered as part of the lesions. All quantifications were performed in a blinded manner. If there were several lesions of one type on one slide, only one lesion was selected for all quantifications. We defined different areas in the non-lesional white matter: PPWM1 was defined as one high power field (HPF) (40X, 0.0625 mm^2^ per HPF) away from the lesion border (corresponding to a distance of 0.25–0.5 mm from the lesion border) and PPWM2 as three HPFs away from the lesion border (corresponding to a distance of 1–1.25 mm from the lesion border) (Fig. 1a–i). PPWM3 was defined as white matter with a distance of 0.5–1 cm from the lesion border [27]. For quantification of microglia and oligodendrocytes, a morphometric grid was used. Only cells with a nucleus and a positive staining signal were counted. For small lesions, the complete brain area around the lesion was quantified. For larger lesions, 10 high power fields per region (PPWM1, PPWM2, and PPWM3) were analyzed. The average number was determined and cell numbers per square millimeter were calculated.

Quantification with Bielschowsky’s silver impregnation was performed under 40 × magnification microscope objective with the aid of a morphometric counting grid. Since the differentiation between PPWM1 and PPWM2 in sections stained by Bielschowsky’s silver stain was not always feasible, we decided to quantify the axonal density in PPWM1/2 (between 1 and 3 HPFs away from the lesion border). Additionally, axonal densities were determined in control tissue samples, intralesional, and PPWM3. 10 HPFs per region of interest were quantified. The average number of axons that intersected 81 possible grid crossing points was determined.

### Statistical analyses

All statistical analyses were performed with GraphPad Prism 8 or R (v4.1.2). For comparisons with respect to lesion type, each lesion was included into the statistical analyses. For all other comparisons, the mean value of all lesions per patient was determined and used for statistical analyses. For comparison of two groups, Student´s two-tailed *t-test* was applied. Linear regression analysis was performed to determine correlations. When three or more groups were compared to each other, Bonferroni-corrected one-way ANOVA was performed. For all analyses, *p* < 0.05 was considered to be significant (*p < 0.05, **p < 0.01, ***p < 0.001, ****p < 0.0001). For classification and regression tree analysis, the CRAN package *rpart* (v4.1.16) was used. The focus of the analysis was to determine the factor with the greatest influence on the average cell number of activated microglia in PPWM1, PPWM2, and PPWM3. Age and sex of the individual patients, cohort, and lesion types were included as predictors. To determine whether the included factors influence each other, a linear mixed model was used. A meta-analysis using the CRAN package *meta* (v6.1) was used to investigate whether individual patient heterogeneity or lesion types were a more decisive factor [3].

## Results

We quantified the numbers of microglia and oligodendrocytes in MS autopsy tissue samples in a cohort of 32 patients and 37 control white matter tissue samples. We analyzed 96 MS tissue blocks including 9 active lesions, 34 mixed, and 57 inactive lesions [25]. We focused our analyses in MS tissue samples on the non-lesional white matter, either PPWM1, PPWM2 or PPWM3 (Fig. 1a–i).

### HLA-DR + microglia numbers are highest in PPWM1 around active and mixed lesions

We quantified the numbers of microglia using IHC for HLA-DR, CD68, and P2RY12 (Fig. 1a–i). HLA-DR and CD68 are some of the most widely used makers to study microglia and blood derived macrophages in human CNS tissue samples, whereas P2RY12 is preferentially expressed by homeostatic microglia [40]. Since we did not observe major differences in microglia numbers between the Münster and the NBB cohort, we decided to pool the data from both cohorts (Supplementary Fig. S1a). We observed significantly higher numbers of HLA-DR + and CD68 + microglia in PPWM1 compared to PPWM2 and PPWM3 (Fig. 1j, k). Numbers of P2RY12 + microglia were higher in PPWM3 and control samples compared to PPWM2 (Fig. 1l). Since HLA-DR labeled more microglia cells than CD68 or P2RY12, we decided to focus our subsequent analyses on HLA-DR + microglia. When we stratified our cohort with respect to lesion type, we observed the highest densities of HLA-DR + microglia in PPWM1 in all lesion types; however, only around active and mixed lesion we observed significantly higher HLA-DR + microglia densities in PPWM1 compared to PPWM2 and/or PPWM3 (Fig. 1m). Numbers of HLA-DR + microglia in PPWM1 and PPWM2 correlated significantly with each other (*r* = 0.78, *p* < 0.0001) (Fig. 1n). Stratification of CD68 + microglia with respect to lesion type provided similar results; however, the densities of CD68 + microglia were in average lower (Supplementary Fig. S1b, c). Numbers of P2YR12 + microglia are lower than numbers of CD68 + or HLA-DR + cells and we observed only few significant differences between PPWM1, PPWM2, and PPWM3 (Supplementary Fig. S1d, e).

### Age has a strong effect on microglia numbers in non-lesional white matter

**Fig. 2:**
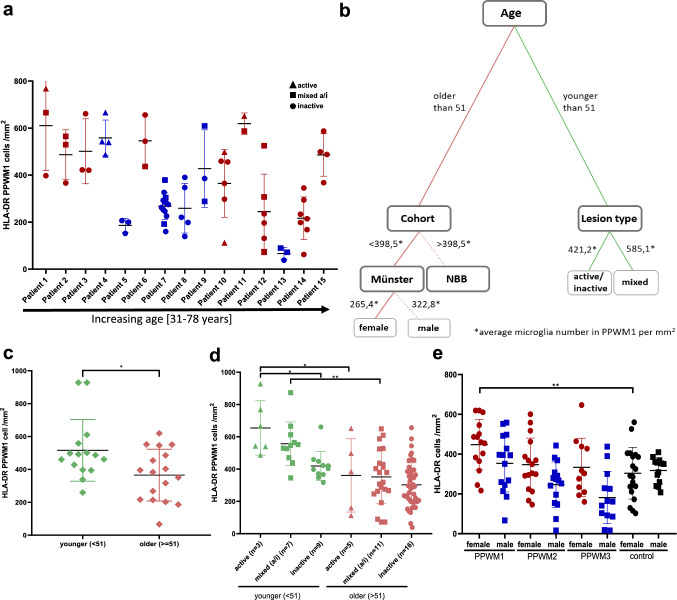
Age has a strong effect on microglia numbers in PPWM1. To analyze the heterogeneity between patients, we selected patients for which three or more lesions were available (*n* = 15). We observed a high heterogeneity in the numbers of HLA-DR-positive microglia in PPWM1 between patients, which was higher than the heterogeneity between lesion types (meta-analysis, *p*-values < 0.0001). Red indicates female patients and blue male patients. The graph depicts the quantification for individual lesions (**a**). Age had the strongest effect on HLA-DR + microglia in PPWM1 as revealed by a classification tree (**b**). This was validated in an independent analysis, demonstrating that patients younger than 51 had higher numbers of HLA-DR-positive microglia compared to older individuals. The graph depicts the average number of HLA-DR + microglia in PPWM1 per patient (**c**). To exclude a bias due to different proportions of lesion types in younger compared to older patients, we stratified the lesions according to lesion type. HLA-DR + densities were higher in active and mixed lesions of younger compared to older patients. The graph depicts microglial numbers per lesion; the number in brackets on the *x*-axis provides the number of individuals (**d**). Quantification of HLA-DR-positive cells shows a trend to higher microglia numbers in PPWM1, PPWM2, and PPWM3 in female compared to male individuals with MS. The graph depicts the average microglia number per patient (**e**). *p < 0.05, **p < 0.01, ***p < 0.001, ****p < 0.0001

Next, we addressed the question whether the heterogeneity of microglial counts between individuals is more prominent than the heterogeneity between different lesion types. For this analysis, we only included patients from which we had three or more lesions available (*n* = 15). Interestingly, heterogeneity was higher between individual patients than differences among lesion types (meta-analysis, *p*-values < 0.0001) (Fig. 2a).

To identify factors contributing to the patient-related heterogeneity in microglial numbers, we performed decision (classification) tree analysis with microglia cell count as the dependent variable and age, cohort, sex, and lesion type as predictors. A generalized linear mixed model was used to account for predictors that are dependent on each other. Since previous analyses had shown that microglia numbers in PPWM1 and PPWM2 are significantly correlated with each other (Fig. 1n), we performed this analysis separately for PPWM1 and PPWM2 (Fig. 2b and Supplementary Fig. S2a). The decision tree identified “age” as the factor with the largest impact on microglial cell counts in PPWM1 and PPWM2 (Fig. 2b and Supplementary Fig. S2a). Patients younger than 51 years showed higher microglia numbers in PPWM1 compared to older patients (*p* < 0.05, Fig. 2b). As second most important influencing factor in PPWM1, “lesion type” played a role in the subgroup of younger patients. Younger patients with a high number of mixed lesions showed the highest overall microglia counts in PPWM1. For patients aged 51 years or older, the decision tree analysis showed that the factor “cohort” had the second largest impact on microglia cell counts in PPWM1. Comparable results were observed when we performed a similar analysis for PPWM2; patients younger than 45 years had a higher number of microglia compared older patients (Supplementary Fig. S2a). Also comparable with the results in PPWM1, "lesion type" was identified as the second most important factor in the subgroup of younger patients in PPWM2 (Supplementary Fig. S2a). Interestingly, in PPWM3, sex had the strongest effect (Supplementary Fig. S2b). Since the decision tree revealed that “age” had the greatest influence on microglial numbers in PPWM1, we validated this relation between age and numbers of HLA-DR-positive cells in an independent analysis of the data, demonstrating that patients below 51 years of age had higher microglia numbers compared to patients above 51 years (Fig. 2c). To determine whether this result was influenced by different proportions of lesion types in younger compared to older patients, we analyzed the proportions of the different lesion types in patients below or above 51 years of age. Indeed, patients below 51 years of age had a higher percentage of active (32% vs. 7% in) and a lower percentage of inactive lesions (36% vs. 64%) compared to patients above 51 years of age. However, when we compared the microglial densities in PPWM1 with respect to lesion type between the two age groups, we observed significantly lower microglia densities in PPWM1 of active and mixed lesions in older compared to younger patients demonstrating that higher age indeed correlated with decreased microglia densities in PPWM1 (Fig. 2d). A similar pattern was observed for PPWM2 and PPWM3; however, the differences did not reach statistical significance (Supplementary Fig. S2c, d).

Since our results from the decision tree analysis suggests that sex may also influences microglial numbers (Fig. 2b, Supplementary Fig. S2a, b), we compared the microglia numbers between male and female patients with MS. For this purpose, the mean microglia numbers in PPWM1, PPWM2, and PPWM3 for each patient independent of the lesion type were determined (Fig. 2e). Female MS patients showed a trend to higher cell density of HLA-DR-positive microglia in PPWM1, PPWM2, and PPWM3 compared with male MS individuals; however, only the difference between female controls and PPWM1 from female MS patients reached statistical significance (Fig. 2e).

### Microglial and oligodendroglial numbers correlate positively in PPWM1

**Fig.3:**
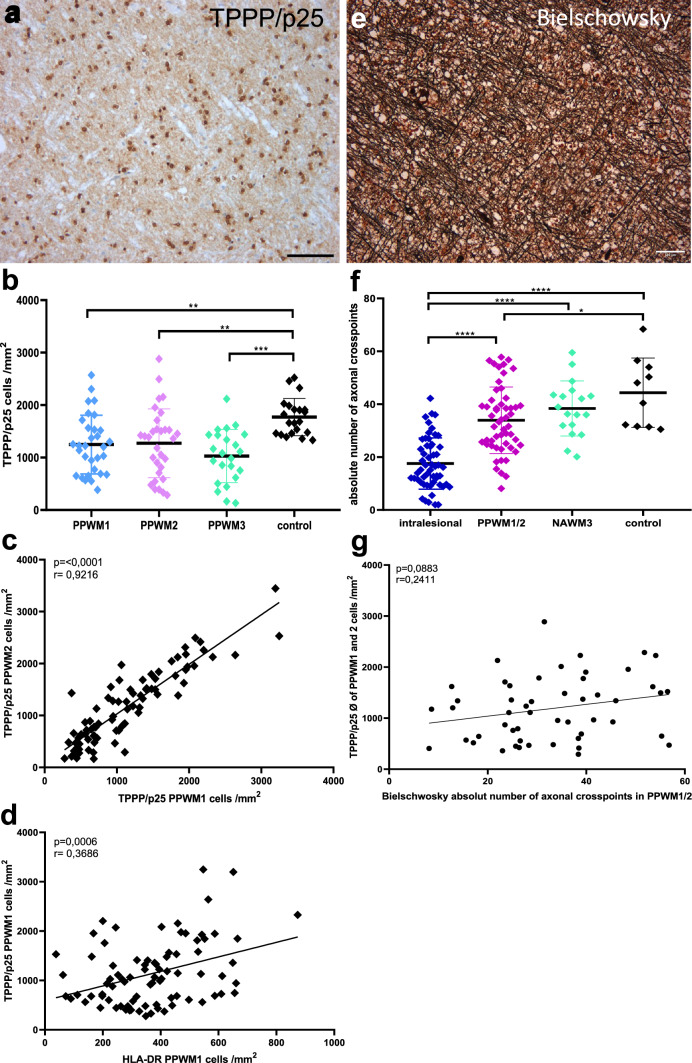
Microglial and oligodendroglial numbers correlate positively in PPWM1. Oligodendrocytes were identified by immunohistochemistry for TPPP/p25 (**a**). We observed significantly lower oligodendroglial numbers in PPWM1, PPWM2, and PPWM3 compared to controls (**b**). We observed a high correlation between the number of TPPP/p25-positive oligodendrocytes in PPWM1 and PPWM2 (**c**). We observed no negative correlation between oligodendroglial and microglial densities (**d**). Axonal densities were determined in sections stained with Bielschowsky's silver stain (**e**). Axonal densities are significantly decreased intralesional and in PPWM1/2 and PPWM3 compared to control tissue samples (**f**). We observed no significant correlation between oligodendroglial and axonal densities in PPWM1/2 and the average of PPWM1 and PPWM2 (**g**). Graph in b depicts the average number of oligodendrocytes per patient; the graphs in **c**, **d**, **f**, and **g** depict cells or axonal densities per lesion. Scale bars: **a** and **e** 100 µm, *p < 0.05, **p < 0.01, ***p < 0.001, ****p < 0.0001

Next, we wanted to determine whether microglia activation correlated with oligodendroglial loss. To quantify the numbers of oligodendrocytes in non-lesional white matter, we performed IHC for the tubulin polymerization-promoting protein (TPPP/p25), which is a microtubule nucleation factor. Anti-TPPP/p25 reliably labels oligodendrocytes in formalin-fixed paraffin-embedded autopsy tissue (Fig. 3a) [21, 22]. We observed significantly lower numbers of oligodendrocytes in PPWM1, PPWM2, and PPWM3 compared to controls, and a high correlation between oligodendroglial numbers in PPWM1 and PPWM2 (*r* = 0.92, *p* < 0.0001), but no significant differences between PPWM1, PPWM2, and PPWM3 (Fig. 3b, c). Interestingly, we found a positive correlation between oligodendroglia and microglial numbers suggesting that microglia activation itself does not contribute to oligodendroglial loss in PPWM1 (*r* = 0.37, *p* = 0.0006) (Fig. 3d). Therefore, we hypothesized that oligodendroglial loss in NAWM may be the consequence of axonal injury and subsequent Wallerian degeneration. Axons were identified using Bielschowsky’s silver stain (Fig. 3e). Since the differentiation between PPWM1 and PPWM2 was not reliably feasible in all sections stained by Bielschowsky’s stain, we decided to determine axonal densities in PPWM1/2 (including PPWM1 and PPWM2), PPWM3, and controls. The axonal density was significantly lower intralesional compared to PPWM1/2, PPWM3, and controls (Fig. 3f); however, we observed no significant correlation between axonal and oligodendroglial densities in PPWM1/2 and the average of PPWM1and PPWM2 (*r* = 0.24, *p* = 0.0883) (Fig. 3g).

## Discussion

Here, we describe that the extent of microglia activation in PPWM in MS patients depends on the proximity to the lesion, lesion type, age, and potentially additional individual factors. In PPWM oligodendroglial and axonal densities were significantly lower than those in control tissue samples; however, we found no negative correlation between microglia and oligodendroglial densities.

So far, only few histological studies have investigated diffuse microglia activation in the non-lesional white matter of MS patients. Increased microglia numbers in NAWM of MS patients compared to control white matter as well as in patients with progressive MS compared to RRMS have been described [27, 37]. In contrast, the number of imaging studies examining diffuse microglia activation in MS patients has significantly increased in recent years. Conventional magnetic resonance imaging (MRI) can be used to detect focal gadolinium enhancing lesions, but it is not sensitive enough to visualize the diffuse pathology in NAWM. Currently, the most sensitive imaging technology to monitor diffuse microglia activation in NAWM is TSPO PET imaging [2]. Radioligands bind to TSPO, which is expressed not only on the outer mitochondrial membrane of activated microglia but also on other glial cells, especially astrocytes [5, 10, 31]. Here, we describe that the extent of microglia activation depends on the proximity to lesions and the lesion type. We observed significantly higher numbers of microglia in the PPWM1 of active and mixed lesions compared to controls, but no significant difference between PPWM1 around inactive lesions and controls. The numbers of microglia cells in PPWM1 and PPWM2 in a given individual correlated closely with each other. This is in line with observations from imaging studies using TSPO PET tracers, which describe a higher signal intensity in perilesional areas compared to NAWM further away as well as a correlation between the signal intensity in T2 lesions and perilesional areas [45]. The observation of higher microglia densities in PPWM1 compared to PPWM2 and/or PPWM3 around active and mixed lesions suggests that soluble inflammatory mediators secreted by myeloid and/or other inflammatory cells present in these two lesion types contribute to increased microglial densities in periplaque white matter. Similar gradients of microglia densities have been observed in normal appearing cortical gray matter and cortical lesions from patients with severe meningeal inflammation as well as in thalamic lesions and normal appearing gray matter adjacent to the ventricular system. In cortical and periventricular gray matter the highest microglia densities were observed close to the CSF spaces with decreasing densities with increasing distance to the CSF spaces [32, 33]. Increased microglia densities in these two CNS regions correlated with neuronal and oligodendroglial loss, suggesting that oligodendroglial and neuronal loss may be either mediated directly by soluble CSF factors or indirectly via microglia. However, the exact inflammatory mediators and underlying pathways are unknown and it remains to be determined whether the same or different mediators are responsible for increased microglia activation adjacent to CSF spaces and active or mixed lesions. The fact that in our study increased microglia densities in PPWM did not correlate inversely with oligodendroglial numbers, which is in contrast to the observations in cortical and periventricular thalamic gray matter lesions, might point to different pathogenic mechanisms.

The heterogeneity in microglia densities between different patients was higher than between different lesion types indicating that individual factors strongly influence the microglia response in patients. These individual factors may include age and sex, but also genetic factors may affect microglia reactions in patients. Interestingly, a study recently published on a preprint server reported molecular oligodendroglial signatures, which were largely shared within an individual across lesions suggesting that individual-dependent factors also influence oligodendroglial pathology [30].

In recent years, age has been identified as a strong modulator of the MS phenotype. Increasing age in MS patients is associated with a reduced relapse rates and response to disease modifying therapies [4, 14, 16, 17]. Interestingly, we identified age as the strongest factor modulating microglia numbers in our cohort of MS post-mortem tissue samples. We observed significantly higher microglia numbers in PPWM1 of younger compared to older MS patients. In MS, younger age at death frequently correlates with shorter disease duration and a more severe disease course, which is usually associated with a higher lesion load and more active lesions. Also in our cohort younger patients displayed a higher percentage of active and a lower percentage of inactive lesions. However, when we compared the microglial densities in the PPWM stratified according to lesion type, we still observed significantly higher HLA-DR + microglia densities in PPWM1 in younger compared to older patients suggesting that higher age is indeed associated with decreased microglia density. This might appear initially counterintuitive, since aging in the healthy rodent brain is associated with a chronic mild inflammation also termed inflammaging [9]. However, aging is also associated with a decreased microglia proliferation and an accumulation of dysfunctional and senescent microglia which could explain our findings [34]. Since age at death in MS is closely interrelated with disease duration, we cannot exclude that not age, but disease duration is a major factor contributing to the decrease of microglia numbers in non-demyelinated white matter. However, since in our cohort the disease duration was only available in a subset of patients, we did not include this parameter in our analyses. Additionally, although we observed lower microglial numbers, it does not exclude the possibility of a more inflammatory and detrimental microglial phenotype in older patients as described in rodent studies [50]. Furthermore, although we ruled out the presence of other neurological diseases based on neuropathological evaluation, we cannot exclude that other comorbidities or medications may have influenced microglia densities within the CNS.

Interestingly, we also observed a trend to higher numbers of microglia in the PPWM of female compared to male patients in our study. To our best knowledge, a correlation of higher microglia numbers in PPWM or NAWM with female sex has not been reported yet, neither in histological nor in PET studies. Significant sex-related differences in microglial numbers, phenotype, and function have been described in embryonic, early postnatal and adult rodents in selected brain areas, indicating that sex, age, anatomical location, and strain-specific differences influence the number of microglia [35, 41, 49]. In line with our observation, increased numbers of microglia in female rodents after LPS injections have been reported, suggesting that microglia in female rat brains react stronger to an inflammatory stimulus. However, the sex differences was only obvious in aged rats [18]. Since our findings are based on a relative low number of patients (15 male vs. 17 female patients), larger histological or imaging studies are required to further validate our findings.

Microglia activation in the NAWM of MS patients is associated with ultrastructural alterations in the nodal and paranodal structures as well as mild changes in the myelin sheath [13, 29, 48, 51]. Furthermore, in the rim of mixed active/inactive lesions which consists mostly of macrophages/microglia, oligodendrocytes are reduced [21]. Therefore, we asked the question whether increased microglial numbers in PPWM and NAWM also affect oligodendrocyte cell numbers. We observed significantly lower numbers of oligodendrocytes in PPWM in MS patients compared to sex- and age-matched controls. Numbers of oligodendrocytes did not correlate inversely with microglia numbers in PPWM, but instead showed a weak positive correlation. This finding suggests that an increase in microglia numbers alone is not sufficient to drive oligodendroglial cell death. However, it also raises the questions whether oligodendrocytes in close proximity to a lesion may acquire a pro-inflammatory phenotype and thereby augment the perilesional inflammatory milieu as suggested for oligodendroglial lineage cells in rodent studies [19, 24]. Oligodendrocytes myelinate up to 50 axonal internodes, and being aware of the high interdependence between oligodendrocytes and axons, we hypothesized that oligodendroglial loss may be the consequence of axonal injury [6, 42, 46]. However, we did not observe a significant correlation between axonal and oligodendroglial densities in PPWM. Although axon densities rose with increasing distance from the lesions, oligodendroglial numbers remained low. Interestingly, in mice mild respiratory COVID was associated with elevated cytokine levels in CSF, increased microglia activation, decreased numbers of mature oligodendrocytes, and impaired neurogenesis for extended time periods. The authors speculate that these neuroinflammatory changes may contribute to cognitive impairment following COVID; however, it cannot be excluded that inflammatory changes in CSF alone may be sufficient to induce oligodendroglia loss [12]. Further studies are required to dissect the molecular mechanisms driving oligodendroglial loss in non-lesional white matter in MS patients as well as the clinical consequences of this oligodendroglial loss.

Our study has limitations. Although we included 96 tissue blocks from 32 patients in this study, our results are still based on a relatively small sample size. To increase our sample size, we included tissue samples from the Netherlands Brain Bank; however, our statistical analyses revealed that also the origin of the cohort may influence the results. Therefore, validation of our results in an independent post-mortem cohort would be desirable. Additionally, although we limited our analyses to lesions within cerebrum and cerebellum, we did not further control for lesion location. In the healthy brain, microglia densities may vary between different anatomical localizations within the cerebrum and cerebellum and we cannot exclude that this also might influence microglia numbers in non-demyelinated white matter in MS.

In summary, we demonstrated that microglia activation in PPWM in MS patients depends on several factors including age, proximity to a lesion, and lesion type. Our findings demonstrate that lesions with persistent focal inflammation also affect PPWM and that age is an important modulator of microglial densities in PPWM.

### Supplementary Information

Below is the link to the electronic supplementary material.

**Supplementary Fig. S1**: Quantification of HLA-DR+ microglia in the Münster and Amsterdam cohort (**a**). Numbers of CD68+ microglia in PPWM1, PPWM2, and PPWM3 around active, mixed, and inactive lesions (**b**). There is a high correlation between CD68+ microglia densities between PPWM1 and PPWM2 (*r* = 0.79, *p* < 0.0001) (**c**). P2RY12+ microglia densities in PPWM1, PPWM2, and PPWM3 around active, mixed, and inactive lesions (**d**). We observed a high correlation between P2RY12+ microglia numbers in PPWM1 and PPWM2 (*r* = 0.79, *p* <0.0001) (**e**). All graphs provide microglia densities per lesion. *p<0.05, **p<0.01, ***p<0.001, ****p<0.0001. **Fig. S2**: Classification tree shows that age has the greatest impact on microglia numbers in PPWM2 (**a**). In PPWM3, sex has the strongest effect on microglia numbers (**b**). MS patients younger than 51 years show a trend to higher HLA-DR+ microglia densities around the different lesion types compared to older patients in PPWM2 (**c**) and PPWM3 (**d**). The graphs in c and d depict the number of microglia per lesion; the numbers in brackets on the *x*-axis provide the number of individuals. (PDF 490 KB)
