# Supplementary material for: Microglia activation in periplaque white matter in multiple sclerosis depends on age and lesion type, but does not correlate with oligodendroglial loss
Source: Acta Neuropathol. 2023 Oct 28;146(6):817–28. doi: 10.1007/s00401-023-02645-2 (PMC10628007; doi:10.1007/s00401-023-02645-2)
Supplement: Supplementary file 1 — Supplementary Fig. S1: Quantification of HLA-DR+ microglia in the Münster and Amsterdam cohort (a). Numbers of CD68+ microglia in PPWM1, PPWM2, and PPWM3 around active, mixed, and inactive lesions (b). There is a high correlation between CD68+ microglia densities between PPWM1 and PPWM2 (r = 0.79, p < 0.0001) (c). P2RY12+ microglia densities in PPWM1, PPWM2, and PPWM3 around active, mixed, and inactive lesions (d). We observed a high correlation between P2RY12+ microglia numbers in PPWM1 and PPWM2 (r = 0.79, p <0.0001) (e). All graphs provide microglia densities per lesion. *p<0.05, **p<0.01, ***p<0.001, ****p<0.0001. Fig. S2: Classification tree shows that age has the greatest impact on microglia numbers in PPWM2 (a). In PPWM3, sex has the strongest effect on microglia numbers (b). MS patients younger than 51 years show a trend to higher HLA-DR+ microglia densities around the different lesion types compared to older patients in PPWM2 (c) and PPWM3 (d). The graphs in c and d depict the number of microglia per lesion; the numbers in brackets on the x-axis provide the number of individuals. (PDF 490 KB) [file 401_2023_2645_MOESM1_ESM.pdf]

Supplementary Figure 1

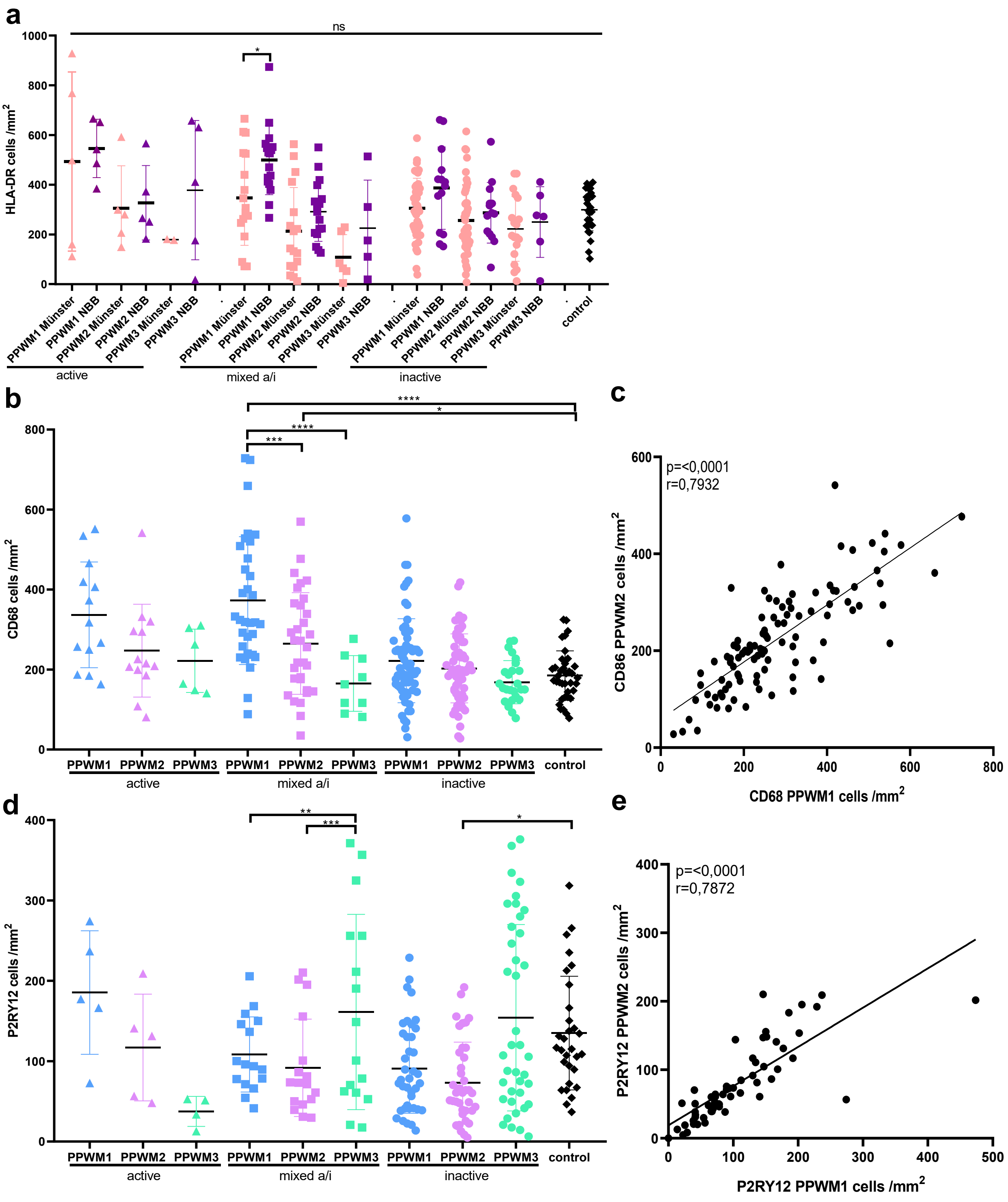

Microglia activation in periplaque white matter in multiple sclerosis depends on age and lesion type, but does not correlate with oligodendroglial loss, Acta Neuropathologica, Wiebke Kessler, tanja.kuhlmann@ukmuenster.de

Supplementary Figure 2

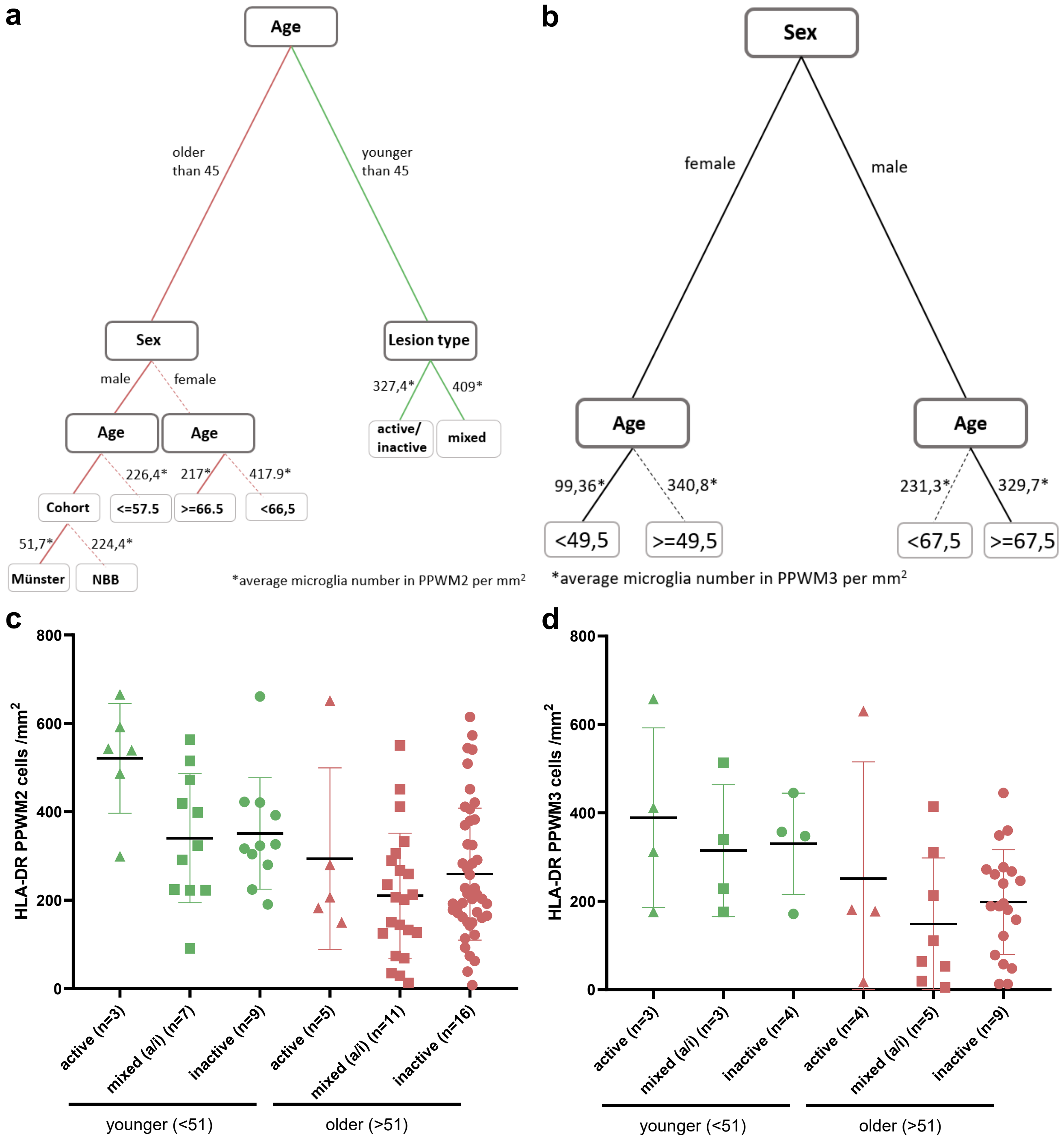

Microglia activation in periplaque white matter in multiple sclerosis depends on age and lesion type, but does not correlate with oligodendroglial loss, Acta Neuropathologica, Wiebke Kessler, tanja.kuhlmann@ukmuenster.de
